# Supplementary material for: UBP12 and UBP13 negatively regulate the activity of the ubiquitin-dependent peptidases DA1, DAR1 and DAR2
Source: eLife. 2020 Mar 25;9:e52276. doi: 10.7554/eLife.52276 (PMC7141810; doi:10.7554/eLife.52276)
Supplement: Transparent reporting form [file elife-52276-transrepform.docx]

***eLife’s* transparent reporting form**

We encourage authors to provide detailed information *within their submission* to facilitate the interpretation and replication of experiments. Authors can upload supporting documentation to indicate the use of appropriate reporting guidelines for health-related research (see [EQUATOR Network](http://www.equator-network.org/%20)), life science research (see the [BioSharing Information Resource](https://biosharing.org/)), or the [ARRIVE guidelines](http://www.plosbiology.org/article/info:doi/10.1371/journal.pbio.1000412) for reporting work involving animal research. Where applicable, authors should refer to any relevant reporting standards documents in this form.

If you have any questions, please consult our Journal Policies and/or contact us: [editorial@elifesciences.org](mailto:editorial@elifesciences.org).

**Sample-size estimation**

- You should state whether an appropriate sample size was computed when the study was being designed
- You should state the statistical method of sample size computation and any required assumptions
- If no explicit power analysis was used, you should describe how you decided what sample (replicate) size (number) to use

Please outline where this information can be found within the submission (e.g., sections or figure legends), or explain why this information doesn’t apply to your submission:

No explicit power analyses were used to determine the sample sizes. In general, around 10 samples within each biological repeat were taken for each macroscopic phenotypical experiment. For molecular experiments, different samples (at least 3 for old time-points and increasing sample sizes for young time-points) were pooled per biological repeat to obtain representative results. These sample sizes are generally accepted in the scientific community and should be sufficient to represent the biological variation for each experiment. Details on the sample sizes can be found in the material and methods section for each experiment, the exact sample sizes and all raw data are detailed in the respective source data files of each experiment.

**Replicates**

- You should report how often each experiment was performed
- You should include a definition of biological versus technical replication
- The data obtained should be provided and sufficient information should be provided to indicate the number of independent biological and/or technical replicates
- If you encountered any outliers, you should describe how these were handled
- Criteria for exclusion/inclusion of data should be clearly stated
- High-throughput sequence data should be uploaded before submission, with a private link for reviewers provided (these are available from both GEO and ArrayExpress)

Please outline where this information can be found within the submission (e.g., sections or figure legends), or explain why this information doesn’t apply to your submission:

For each quantitative experiment, we performed at least three independent biological repeats which were sown and harvested at a different time (material and methods). All source data and statistics (calculations, statistical tests, Post-Hoc tests, p-values) are provided in the source data files linked to the respective figures and a brief description is given in the figure legends. No outliers were excluded from the data analyses.

**Statistical reporting**

- Statistical analysis methods should be described and justified
- Raw data should be presented in figures whenever informative to do so (typically when N per group is less than 10)
- For each experiment, you should identify the statistical tests used, exact values of N, definitions of center, methods of multiple test correction, and dispersion and precision measures (e.g., mean, median, SD, SEM, confidence intervals; and, for the major substantive results, a measure of effect size (e.g., Pearson's r, Cohen's d)
- Report exact p-values wherever possible alongside the summary statistics and 95% confidence intervals. These should be reported for all key questions and not only when the p-value is less than 0.05.

Please outline where this information can be found within the submission (e.g., sections or figure legends), or explain why this information doesn’t apply to your submission:

We mentioned in the figure legends the sample size, statistical method and p-value. In the material and methods, the software for the analysis is listed. In addition, for all quantitative measurements, a Source data file is provided that contains the raw data and the complete output of the statistical test.

(For large datasets, or papers with a very large number of statistical tests, you may upload a single table file with tests, Ns, etc., with reference to sections in the manuscript.)

**Group allocation**

- Indicate how samples were allocated into experimental groups (in the case of clinical studies, please specify allocation to treatment method); if randomization was used, please also state if restricted randomization was applied
- Indicate if masking was used during group allocation, data collection and/or data analysis

Please outline where this information can be found within the submission (e.g., sections or figure legends), or explain why this information doesn’t apply to your submission:

All information can be found in the material and methods section

**Additional data files (“source data”)**

- We encourage you to upload relevant additional data files, such as numerical data that are represented as a graph in a figure, or as a summary table
- Where provided, these should be in the most useful format, and they can be uploaded as “Source data” files linked to a main figure or table
- Include model definition files including the full list of parameters used
- Include code used for data analysis (e.g., R, MatLab)
- Avoid stating that data files are “available upon request”

Please indicate the figures or tables for which source data files have been provided:

Figure 1

Figure 2

Figure 3

Figure 5

Figure 1-figure supplement 2

Figure 1-figure supplement 3

Figure 1-figure supplement 4

Figure 2-figure supplement 1

Figure 2-figure supplement 2

Figure 2-figure supplement 3

Figure 2-figure supplement 4

Figure 2-figure supplement 5

Figure 2-figure supplement 6

Figure 2-figure supplement 7

Figure 2-figure supplement 10

Figure 3-figure supplement 1

Figure 3-figure supplement 2

Figure 3-figure supplement 3

Figure 3-figure supplement 4

Figure 6-figure supplement 1
